# BMSC-Derived Exosomal CircHIPK3 Promotes Osteogenic Differentiation of MC3T3-E1 Cells via Mitophagy

**DOI:** 10.3390/ijms24032785

**Published:** 2023-02-01

**Authors:** Shaoyang Ma, Sijia Li, Yuchen Zhang, Jiaming Nie, Jiao Cao, Ang Li, Ye Li, Dandan Pei

**Affiliations:** Key Laboratory of Shaanxi Province for Craniofacial Precision Medicine Research, College of Stomatology, Xi’an Jiaotong University, Xi’an 710000, China

**Keywords:** osteogenesis, exosome, mitophagy, circRNA, stem cells

## Abstract

Exosome-based therapy is emerging as a promising strategy to promote bone regeneration due to exosomal bioactive cargos, among which circular RNA (circRNA) has recently been recognized as the key effector. The role of exosomal circRNA derived from bone marrow mesenchymal stem cells (BMSCs) has not been well-defined. The present study aimed to clarify the regulatory function and molecular mechanism of BMSC-derived exosomal circRNA in osteogenesis. Exosomes derived from bone marrow mesenchymal stem cells (BMSC-Exos) were isolated and identified. BMSC-Exos’ pro-osteogenic effect on MC3T3-E1 cells was validated by alkaline phosphatase (ALP) activity and Alizarin Red staining. Through bioinformatic analysis and molecular experiments, circHIPK3 was selected and verified as the key circRNA of BMSC-Exos to promote osteoblast differentiation of MC3T3-E1 cells. Mechanistically, circHIPK3 acted as an miR-29a-5p sponge and functioned in mitophagy via targeting miR-29a-5p and PINK1. Additionally, we showed that the mitophagy level of MC3T3-E1 cells were mediated by BMSC-Exos, which promoted the osteogenic differentiation. Collectively, our results revealed an important role for BMSC-derived exosomal circHIPK3 in osteogenesis. These findings provide a potentially effective therapeutic strategy for bone regeneration.

## 1. Introduction

Effective bone regeneration therapy remains in huge clinical demands at present [1]. Recently, bone marrow mesenchymal stem cells (BMSCs) have attracted broad attention in promoting bone regeneration for their multidirectional differentiation potential [2,3]. However, growing researches have demonstrated that the effectiveness of BMSC therapy depends on paracrine factors, particularly exosomes [4,5]. As a subclass of membrane-coated extracellular vesicles (EVs) with sizes of 30–150 nm, exosomes are produced by almost all cell types of the human body and contain functional cargos [6]. Previous studies have confirmed that exosomes derived from BMSC (BMSC-Exos) could regulate osteoblast differentiation through exosomal bioactive cargos [7,8,9], suggesting exosomes as a promising cell-free strategy in bone regeneration.

In the last decade, circular RNA (circRNA), a newly identified noncoding RNA, has emerged as an essential regulator of osteogenesis [10]. CircRNAs are generated via back-splicing and characterized by covalently closed loop structures [11], which makes them more resistant to RNA exonuclease and more stable in cells [12,13]. Consequently, circRNA may be a more suitable target for clinical therapeutic compared with linear RNAs. Recently, the role of circRNAs in osteogenesis has gained increasing attention. By high-throughput sequencing and prediction, some researchers have reported that the expressions of circRNAs were significantly changed during osteogenic differentiation [14,15], suggesting a close relationship between circRNAs and osteogenesis. More recently, the mechanism of circRNAs in osteogenesis has been discovered. Several circRNAs were reported to interact with osteogenesis-associated microRNAs (miRNAs) through a competitive endogenous RNA (ceRNA) mechanism, which affect the osteogenic differentiation of mesenchymal stem cells [16,17,18]. Additionally, circRNAs have been identified to be enriched and stabile in exosomes and to play multiple biological functions [19]. However, the molecular mechanisms of BMSC-derived exosomal circRNA in regulating osteogenesis is still not well understood, which prompted us to study the deep mechanism.

Mitophagy, selective autophagy of dysfunctional or superfluous mitochondria, is related with many cellular biological functions [20]. Our previous study found that PTEN induced putative kinase 1 (PINK1)/Parkin-mediated mitophagy participated in the osteogenic differentiation of dental pulp stem cells by assisting mineral precursor transfer [21]. Subsequently, the essential role of mitophagy in osteogenesis was confirmed by other studies [22,23,24,25]. Moreover, BMSC-Exos were reported to have the potential of regulating mitophagy in osteoarthritis and renal ischemia/reperfusion injury recently [26,27]. Whether and how BMSC-Exos influences osteogenic differentiation by regulating mitophagy is unclear and will be investigated in the present study. Our data will provide the evidence of BMSC-derived exosomal circRNA as a potential therapeutic target for bone regeneration via cell-free exosome-based strategy.

## 2. Results

### 2.1. BMSC-Exos Enhances Osteogenic Differentiation of MC3T3-E1 Cells

**Figure 1:**
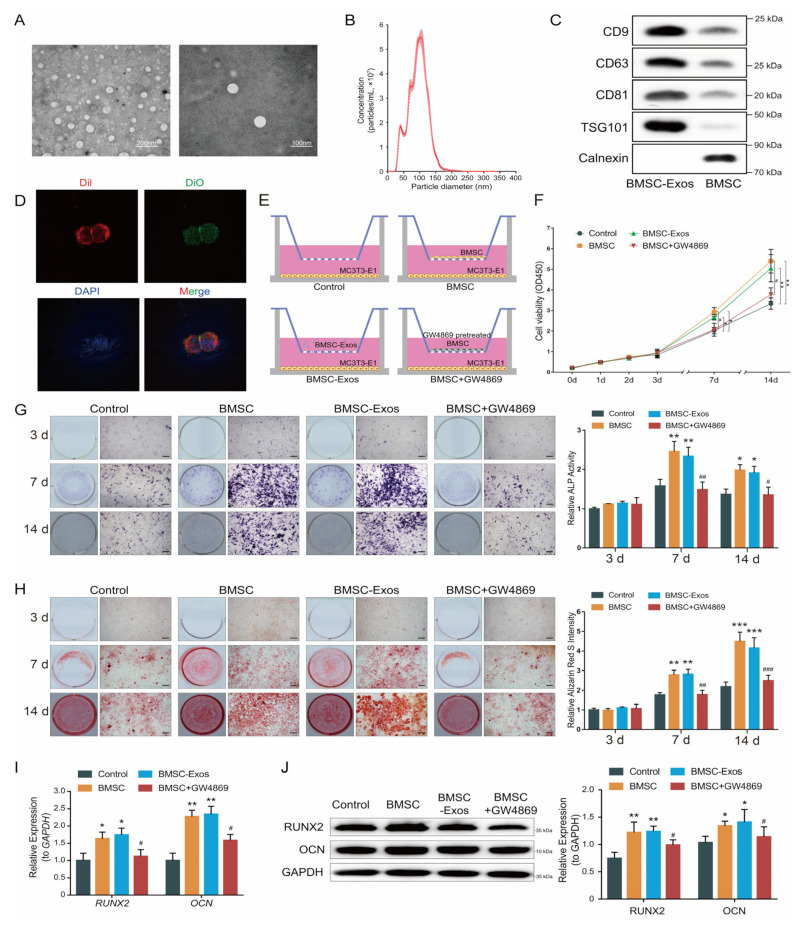
BMSC-Exos promoted osteogenesis. (**A**) Representative picture of BMSC-Exos collected by ultracentrifugation. (**B**) Size distribution profile of BMSC-Exos (*n* = 3). The dots represent the mean value, and the shading represents the SD. (**C**) Western blot analysis of exosome-specific markers (CD9, CD63, CD81 and TSG101) and negative marker (Calnexin). (**D**) Intercellular trafficking of BMSC-Exos. Cytomembranes were labeled by Dil (red); BMSC-Exos were labeled by DiO (Green); nuclei were labeled by DAPI (blue). (**E**) Schematic drawing of the Transwell protocol. (**F**) Cell proliferation assays. Each point represents the mean value from three independent samples. * *p <* 0.05; ** *p <* 0.01. (**G**) ALP staining (left) was examined and activity of ALP (right, *n* = 6) was analyzed by ALP activity assay kit. Bar 100 μm. (**H**) ARS staining was performed (left) and quantified using a microplate reader at 562 nm (right, *n* = 6). Bar: 100 μm. (**I**) Fold changes in the expression of RUNX2 and OCN in MC3T3-E1 cells relative to GAPDH (*n* = 6). (**J**) Western blot analysis (left) and quantification of Western blot results (right, *n* = 3). * *p <* 0.05, ** *p <* 0.01, *** *p <* 0.001 vs. the control group; # *p <* 0.05, ## *p <* 0.01, ### *p <* 0.001 vs. the BMSC group.

The phenotypes of BMSCs were identified by flow cytometric analysis (Figure S1A,B). Then, we extracted BMSC-Exos from the supernatant of the third passage BMSCs. Observation of BMSC-Exos by transmission electron microscope (TEM) revealed that most of the exosomes had a classic round or elliptical shape (Figure 1A). Nanoparticle tracking analysis (NTA) result indicated that the size of BMSC-Exos was generally distributed between 50 nm and 150 nm (Figure 1B). Additionally, Western blot results (Figure 1C) showed that exosome-specific markers (CD9, CD63, CD81 and TSG101) were detected in BMSC-Exos, with no detection of endoplasmic reticulum marker (Calnexin), indicating the successful isolation of the exosomes with high purity.

In order to evaluate the internalization efficiency, BMSC-Exos and MC3T3-E1 cells were labeled with fluorescent carbocyanine dyes CM-Dio (green) and CM-Dil (red), respectively. After co-incubation for 24 h, the internalization efficiency was measured by the laser confocal microscope. The result indicated that the BMSC-Exos had been successfully internalized by the MC3T3-E1 cells (Figure 1D).

As the exosome can readily cross the Transwell chamber membrane [28], we next assess the effect of BMSC-Exos on MC3T3-E1 proliferation and osteogenic differentiation by Transwell coculture system (Figure 1E). Cell counting kit-8 (CCK-8) assays showed that coculture of BMSCs or BMSC-Exos significantly enhanced MC3T3-E1 cell proliferation compared with control groups (Figure 1F). Then, we used GW4869, an inhibitor of exosome secretion [29], to inhibit BMSC-Exos secretion. The GW4869 treatment significantly reduced the exosome amount (Figure S2) and abrogated the functions of BMSC-Exos in promoting cell proliferation (Figure 1F). Then, we tested the pro-osteogenic effect of BMSCs and BMSC-Exos. We detected ALP activity, an important marker of early stage osteogenesis, by both ALP staining and an ALP activity assay. Compared with the control group, greater ALP activity was observed in the BMSC and BMSC-Exos groups on 7 d and 14 d, whereas GW4869 treatment abolished the pro-osteogenic effect (Figure 1G). Similar results were observed with Alizarin Red S staining of calcium deposits (Figure 1H). Consistent with the above results, qRT-PCR and Western blot analysis also revealed an up-regulation of osteogenic markers (RUNX2 and OCN) after BMSC and BMSC-Exos coculture, but not in the BMSC + GW4869 group (Figure 1I,J). The foregoing suggested that BMSCs and BMSC-Exos could enhance osteogenic differentiation of MC3T3-E1 cells, and the pro-osteogenic function of BMSC was dependent on BMSC-Exos.

### 2.2. CircHIPK3 Is Enriched in BMSC-Exos

**Figure 2:**
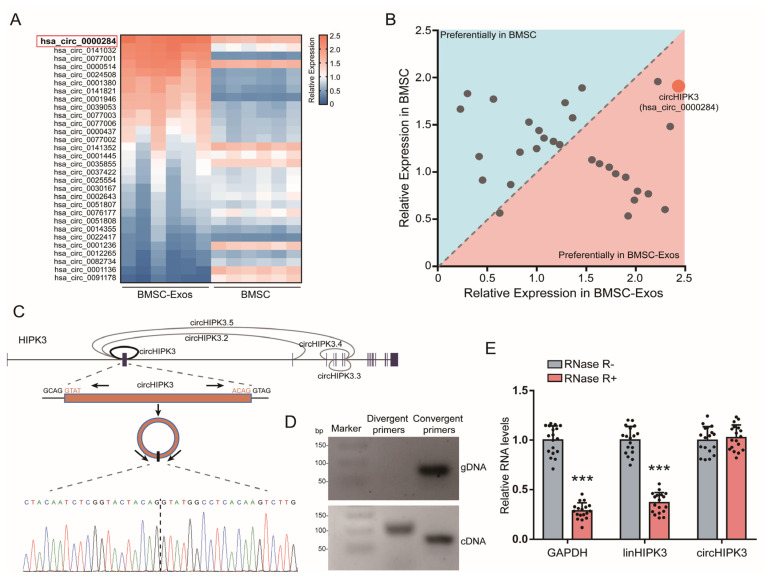
CircHIPK3 was highly expressed in BMSC-Exos. (**A**) Heatmap of highly expressed circRNAs in BMSCs and BMSC-Exos. The color from blue (low) to red (high) represents expression levels of circRNAs. Each row in the map represents a sample and each column represents a gene. The red box represents circHIPK3 (circBase name: hsa_circ_0000284). (**B**) The relative levels of highly expressed circRNAs in BMSCs and BMSC-Exos. The relative expression in BMSCs (Y-axis), against those in BMSC-Exos (X-axis). (**C**) Schematic presentation of circHIPK3 location and the junction site sequence. (**D**) Agarose gel electrophoresis analysis of circHIPK3 PCR products of divergent primer and convergent primer. cDNA: complementary DNA, gDNA: genomic DNA. (**E**) The RNase R digestion resistance test confirmed the stability of circHIPK3 (*n* = 18). *** *p <* 0.001.

Increasing evidence has indicated the function of circRNAs in pro-osteogenic function [10]. To examine possible BMSC-Exos circRNAs involved in promoting osteogenic differentiation of MC3T3-E1 cells, we selected 30 highly expressed circRNAs in BMSC from published data [30,31,32] and the circBase database (http://www.circbase.org/ (accessed on 25 August 2017)). The expressions of these 30 circRNAs in BMSCs and BMSC-Exos were detected by qRT-PCR (Figure 2A). Then, the circHIPK3 (circBase name: hsa_circ_0000284), which had the highest expression level in BMSC-Exos, was selected as a candidate target for further analyses (Figure 2B). 

Based on the data of the UCSC Genome Browser (http://genome.ucsc.edu/ (accessed on 3 February 2014)), circHIPK3 is generated from back-splicing of the second exons of the HIPK3 gene with a length of 1099 nt (Figure 2C). The back-splice junction site of circHIPK3 was amplified using divergent primers and was confirmed by Sanger sequencing (Figure 2C,D). To further verify the covalently closed continuous loop structure of circHIPK3, we designed the divergent and convergent primers to amplify circHIPK3 and its linear form. The agarose gel electrophoresis analysis showed that circHIPK3 could only be amplified from cDNA, while its linear form could be amplified from cDNA and gDNA (Figure 2D). Moreover, we treated BMSCs with RNase R (an exoribonuclease that specifically degrades linear, but not circular or lariat RNAs [33]) and found that circHIPK3 was more stable than its linear form and GAPDH (Figure 2E). In summary, these results indicated that circHIPK3 was with the continuous loop structure and enriched in BMSC-Exos, suggesting that it might be involved in the pro-osteogenic function of BMSC-Exos.

### 2.3. CircHIPK3 Is Critical for Pro-Osteogenic Effect of BMSC-Exos

**Figure 3:**
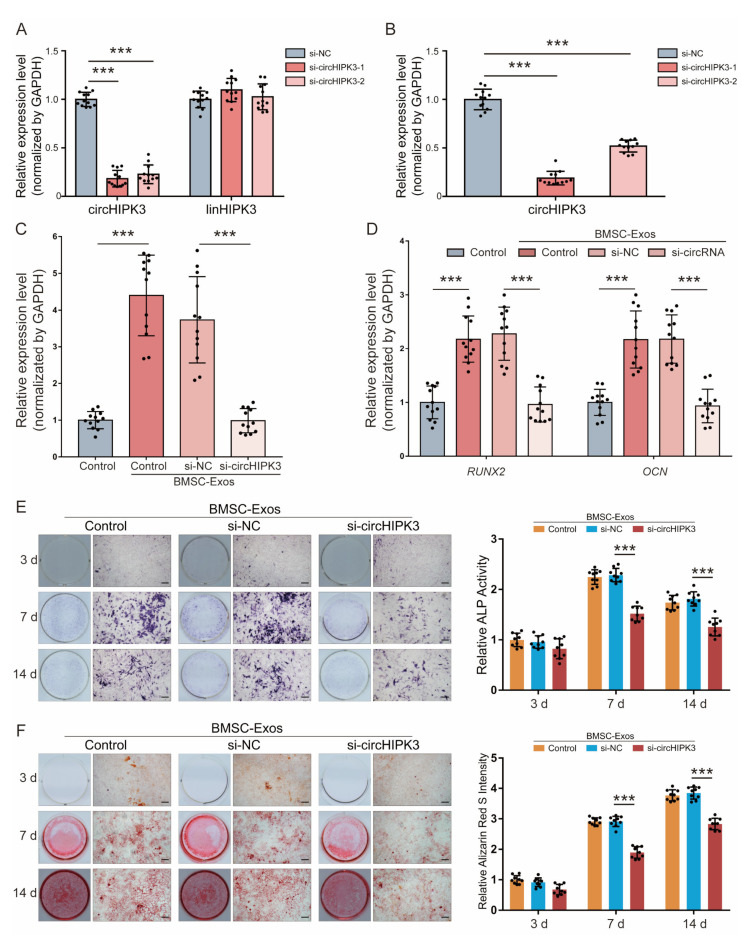
CircHIPK3 promoted osteogenic differentiation of MC3T3-E1 cells. (**A**) circHIPK3 levels and linear HIPK3 in BMSCs were measured by qRT-PCR after transfection with si-NC, si-circHIPK3-1 or si-circHIPK3-2. (**B**) circHIPK3 levels in exosomes derived from BMSCs treated with si-NC, si-circHIPK3-1 or si-circHIPK3-2 were measured by qRT-PCR. (**C**,**D**) circHIPK3 levels (**C**) and RUNX2 and OCN levels (**D**) in MC3T3-E1 cells treated with DMSO (control, *n* = 12) or exosomes derived from BMSCs treated DMSO (BMSC-Exos-control, *n* = 12), with si-NC (BMSC-Exos-si-NC, *n* = 12) or si-circHIPK3-1 (BMSC-Exos-si-circHIPK3, *n* = 12). *** *p* < 0.001. (**E**) ALP staining (left) was examined and activity of ALP (right, *n* = 6) was analyzed by ALP activity assay kit. Bar 100 μm. (**F**) ARS staining was performed (left) and quantified using a microplate reader at 562 nm (right, *n* = 6). Bar: 100 μm. *** *p <* 0.001.

We studied the effect of BMSC-Exos circHIPK3 on the osteogenic differentiation of MC3T3-E1. Two small interfering RNA oligos (si-circHIPK3-1 and si-circHIPK3-1) were designed to target the junction site of circHIPK3. The qRT-PCR result showed that both of the two siRNAs could efficiently downregulate circHIPK3 expression in BMSCs and BMSC-Exos compared with the si-NC (Figure 3A,B), while the expression of its linear form did not change significantly (Figure 3A). We selected si-circHIPK3-1 for subsequent experiments because it was the more effective siRNA. Compared with the MC3T3-E1 cells treated with exosomes derived from untreated BMSCs or si-NC pretreated BMSCs, the expressions of circHIPK3 was significantly downregulated in the MC3T3-E1 cells treated with exosomes from BMSCs with si-circRNAs (Figure 3C).

**Figure 4:**
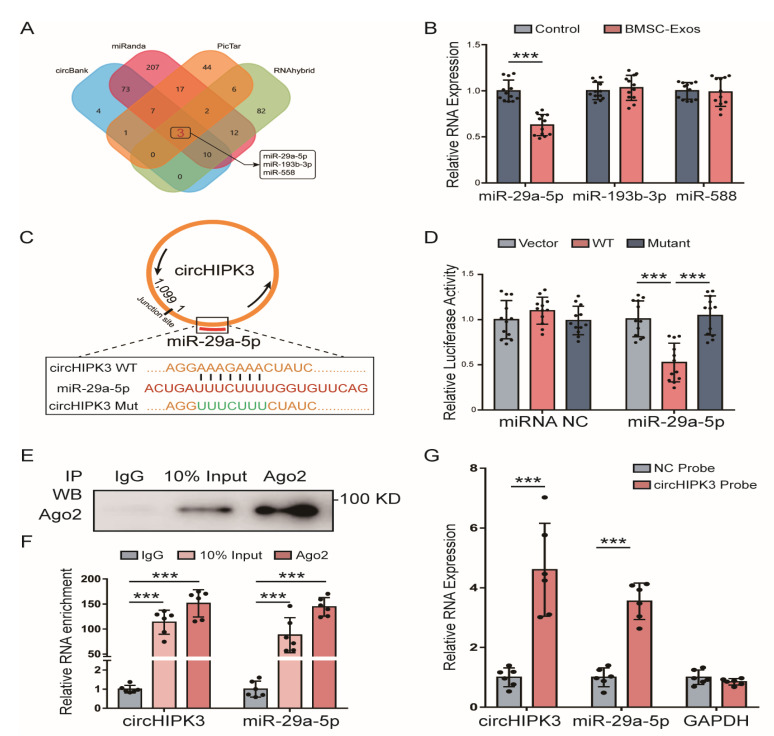
CircHIPK3 served as a miRNA sponge of miR-29a-5p. (**A**) Three miRNAs (miR-29a-5p, miR-193b-3p and miR-558) were predicted to be candidate targets of circHIPK3. (**B**) Expressions of miR-29a-5p, miR-193b-3p and miR-558 in MC3T3-E1 cells treated with BMSC-Exos (*n* = 12). (**C**) Schematic representation of circHIPK3 with the predicted target site for miR-29a-5p. The mutant site of circHIPK3 is indicated (green). (**D**) Luciferase reporter analysis was performed to examine the binding ability between miR-29a-5p and circHIPK3. Reporter constructs containing either circHIPK3-WT or circHIPK3-Mut at the predicted miR-29a-5p target sequences were co-transfected into HEK293T cells, along with miR-29a-5p or miRNA NC mimics (*n* = 12). (**E**,**F**) RIP experiments were performed using the Ago2 antibody (E) and specific primers were used to detect the enrichment of circHIPK3 and miR-29a-5p in MC3T3-E1 ((**F**), *n* = 6). *** *p* < 0.001. (**G**) Biotinylated-probe pull-down assay for circHIPK3 and miR-29a-5p by qRT-PCR in MC3T3-E1 cells (*n* = 6). The probe was designed according to the junction region of circHIPK3. *** *p* < 0.001.

The pro-osteogenic effect of exosomes derived from BMSCs transfected with si-circHIPK3 was detected. Relative mRNA expression levels of osteoblast differentiation markers (RUNX2 and OCN) were significantly increased in MC3T3-E1 cells treated with BMSC-Exos or si-NC BMSC-Exos for 14 days, while the treatment of si-circHIPK3 BMSC-Exos abolished the increase (Figure 3D). As shown in Figure 4E, knockdown of circHIPK3 significantly decreased the osteogenic differentiation of MC3T3-E1 cells, as indicated by ALP staining and activity. The same conclusions were reached by the intensity of Alizarin red staining (Figure 3F). Together, these data indicate that knockdown of circHIPK3 impaired the pro-osteogenic effect of BMSC-Exos.

### 2.4. CircHIPK3 Directly Binds to miR-29a-5p

Since circRNAs can function as miRNA sponges to bind to functional miRNAs and then regulate gene expression, we examined the potential miRNAs associated with circHIPK3. By using the tool of circBank, miRanda, PicTar and RNAhybrid to predict miRNAs that could bind with the circHIPK3 sequence, we obtain three miRNAs (miR-29a-5p, miR-193b-3p and miR-558) as candidates (Figure 4A). Among these miRNAs, only the level of miR-29a-5p was decreased in MC3T3-E1 cells treated with BMSC-Exos (Figure 4B), which suggested that circHIPK3 could interact with miR-29a-5p. Therefore, we selected miR-29a-5p for further investigation.

The specific binding site between circHIPK3 and miR-29a-5p was predicted using the StarBase (http://starbase.sysu.edu.cn (accessed on 24 January 2022) (Figure 4C). The dual-luciferase reporter assay showed that the luciferase signal of the circHIPK3 reporter was suppressed by miR-29a-5p, whereas the introduction of mutations in the circHIPK3 abolished the inhibitory effect, which confirmed that circHIPK3 could directly interact with miR29a-5p (Figure 4D). Additionally, the Ago2-RNA-immunoprecipitation (Ago2-RIP) assay with anti-Ago2 antibody and IgG antibody was performed to investigate the circRNA-miRNA binding. As expected, the expression levels of circHIPK3 and miR-29a-5p pulled down by anti-Ago2 were significantly higher than those in the anti-IgG group (Figure 4F). Next, by using the probe targeting circHIPK3 junction site, we found that circHIPK3 and miR-29a-5p were significantly more abundant compared with the control group (Figure 4G). Taken together, the above data confirmed that circHIPK3 functioned as a miR-29a-5p sponge.

### 2.5. CircHIPK3 Promotes Mitophagy of MC3T3-E1 Cells through miR-29a-5p/PINK1 Axis

**Figure 5:**
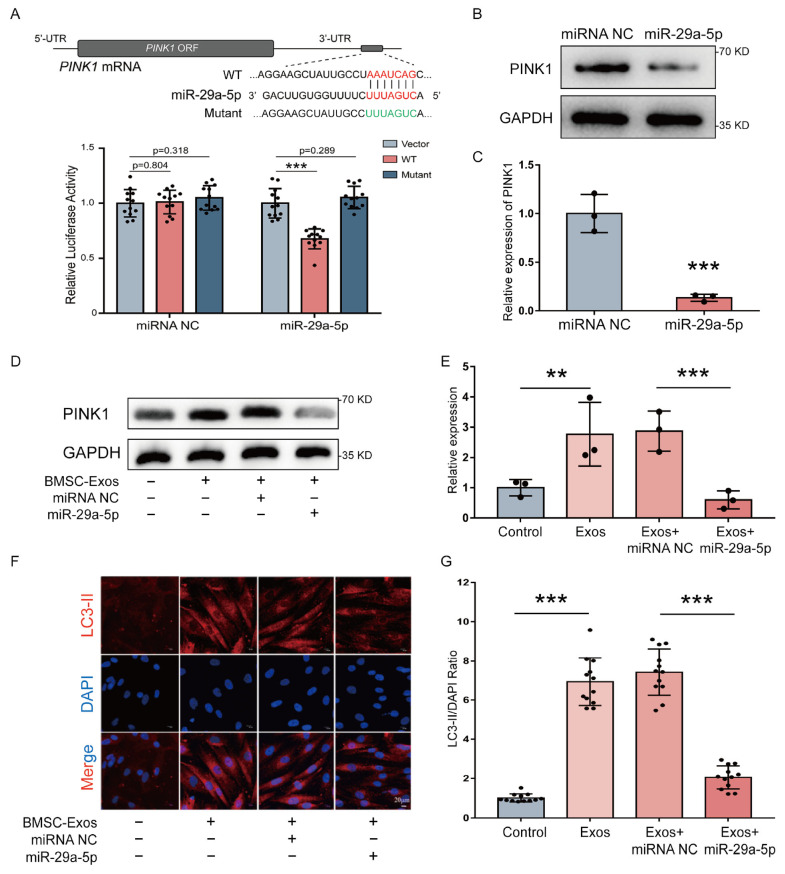
CircHIPK3 promoted mitophagy of MC3T3-E1 cells through miR-29a-5p/PINK1 axis. (**A**) Schematic representation of PINK1 3′-UTR with the predicted target site for miR-29a-5p. The mutant site of PINK1 3′-UTR is indicated (green). Luciferase reporter analysis was performed to examine the binging ability between miR-29a-5p and PINK1 3′-UTR. Reporter constructs containing either PINK1 3′-UTR-wt or PINK1 3′-UTR-mut at the predicted miR-29a-5p target sequences were co-transfected into HEK293T cells, along with miR-29a-5p or miRNA NC mimics (*n* = 12). (**B**,**C**) Western blot assay and quantification of PINK1 in MC3T3-E1 cells transfected with miR-29a-5p or miRNA NC mimics (*n* = 3). *** *p* < 0.001. (**D**,**E**) Western blot assay and quantification of PINK1 in MC3T3-E1 cells treated with PBS (control), BMSC-Exos (Exos), BMSC-Exos and miRNA NC (Exos + miRNA NC) or BMSC-Exos and miR-29a-5p mimic (Exos + miR-29a-5p) (*n* = 3). ** *p* < 0.01, *** *p <* 0.001. (**F**,**G**) Representative LC3 immunofluorescence staining (LC3 stained with red and nuclei stained with blue) and quantitative results of LC3 intensity (*n* = 12). *** *p* < 0.001. Bar: 20 μm.

We used miRanda, miRBridge and TargetScan to predict potential target genes of miR-29a-5p and identified PINK1 gene as a potential target of miR-29a-5p (Figure 5A). To identify whether miR-29a-5p directly targets the 3′-UTR of PINK1 gene, WT-PINK1 or Mutant-PINK1 was co-transfected with miR-29a-5p mimics into MC3T3-E1 cells. The dual-luciferase assay showed that the luciferase activity of WT-PINK1 was significantly inhibited by miR-29a-5p mimics, while Mutant-PINK1 relieved the effect induced by miR-29a-5p (Figure 5A). Then, protein expressions of PINK1 transfected with miR-29a-5p mimics or miRNA NC were determined by Western blot analysis (Figure 5B). The results showed that protein expression of PINK1 was significantly downregulated by miR-29a-5p mimics (Figure 5C). These findings suggested that PINK1 is a direct target gene of miR-29a-5p.

As circHIPK3 was enriched in BMSC-Exos and could sponge miR-29a-5p, we detected the effect of BMSC-exosomal circHIPK3 on PIKN1. The protein expression of PIKN1 was increased by the treatment with BMSC-Exos, which could be abolished by miR-29a-5p overexpression (Figure 5D,E). PINK1 is well known as a pro-mitophagic gene [34]. Therefore, we wondered whether BMSC-Exos could regulate the mitophagy through miR-29a-5p. We treated MC3T3-E1 cells with BMSC-Exos for 14 days and performed LC3 immunofluorescence staining to detect the change of mitophagy level (Figure 5F). The result showed that the mitophagy level was improved by the BMSC-Exos treatment, while miR-29a-5p overexpression reduced the mitophagy level (Figure 5G). Collectively, these results suggest that circHIPK3 promoted PINK1 expression and mitophagy level by competitively binding to miR-29a-5p in MC3T3-E1 cells.

### 2.6. PINK1/Parkin-Mediated Mitophagy Contributed to the Pro-Osteogenic Effect of BMSC-Exos

**Figure 6:**
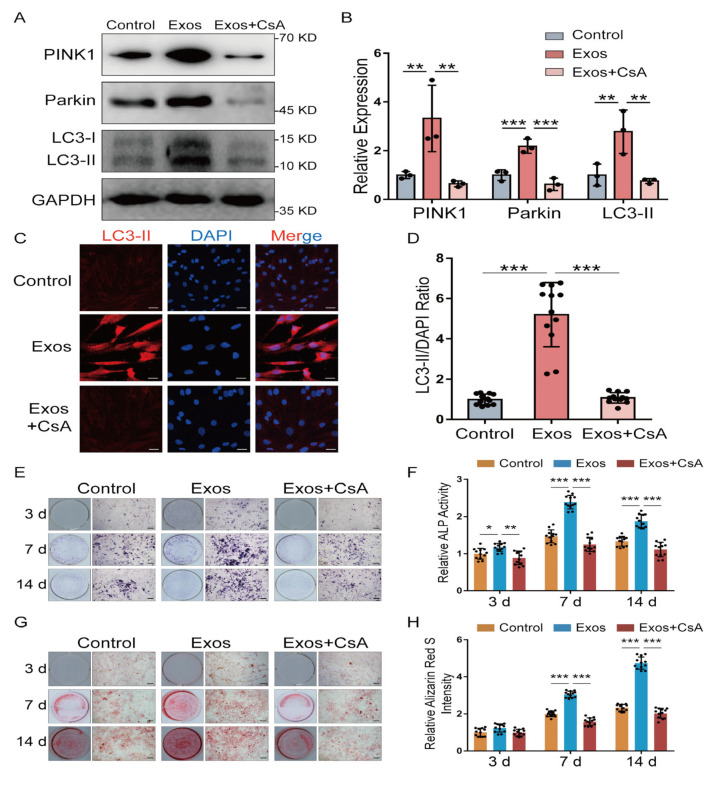
BMSC-Exos promoted osteogenic differentiation of MC3T3-E1 cell through mitophagy. (**A**,**B**) Western blot assay and quantification of osteogenesis-related genes (PINK1, Parkin and LC3-I/II) in MC3T3-E1 cells treated with PBS (Control), BMSC-Exos (Exos) or BMSC-Exos and CsA (Exos + CsA) (*n* = 3). ** *p* < 0.01; *** *p* < 0.001. (**C**,**D**) Representative LC3 immunofluorescence staining (LC3 stained with red and nuclei stained with blue) and quantitative results of LC3 intensity (*n* = 12). *** *p* < 0.001. Bar: 10 μm. (**E**,**F**) ALP staining was examined and activity of ALP was analyzed by ALP activity assay kit (*n* = 6). * *p* < 0.05; ** *p* < 0.01; *** *p* < 0.001. Bar 100 μm. (**G**,**H**) ARS staining was performed and (H) quantified using a microplate reader at 562 nm (*n* = 6). *** *p* < 0.001. Bar: 100 μm.

As PINK1/Parkin-mediated mitophagy was proven to enhance osteogenesis by our previous study [17], we wondered whether mitophagy participates in the pro-osteogenic effect of BMSC-Exos. The expressions of mitophagy-related proteins (PINK1, Parkin and LC-3II) were detected by Western blot (Figure 6A). BMSC-Exos treatment increased the expression of PINK1, Parkin and LC-3II, while the mitophagy inhibitor cyclosporine A (CsA) could significantly eliminate this increase (Figure 6A,B). The result of LC3 immunofluorescence staining also showed that BMSC-Exos could increase mitophagy of MC3T3-E1 cells (Figure 6C,D). Then, we detected the effect of BMSC-Exos-mediated mitophagy on the osteogenesis of MC3T3-E1 cells. The results of ALP staining and the activity assay showed that the pro-osteogenic effect of BMSC-Exos was reduced by mitophagy inhibitor CsA (Figure 6E,F). Meanwhile, Alizarin Red S staining of calcium deposits was performed and yielded similar results (Figure 6G,H). In summary, BMSC-Exos could increase mitophagy of MC3T3-E1 cells, which contributed to the pro-osteogenic effect of BMSC-Exos.

## 3. Discussion

**Figure 7:**
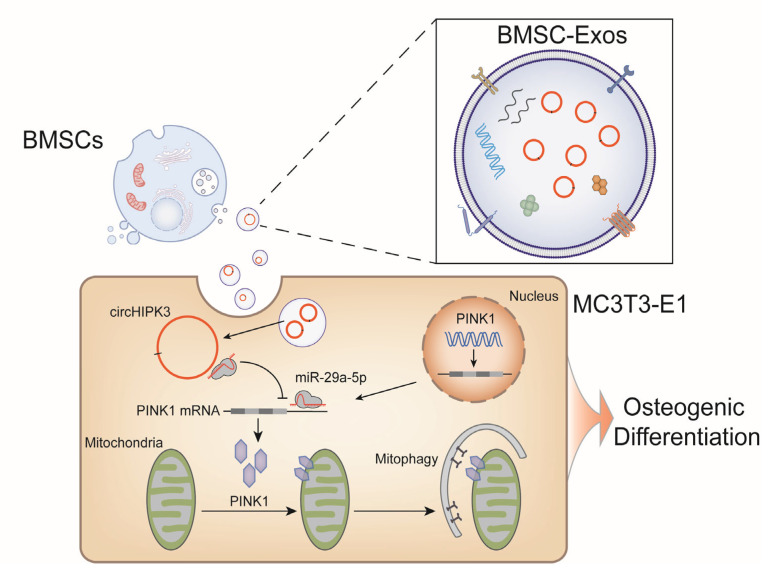
CircHIPK3 is enriched in BMSC-Exos and promotes osteogenic differentiation of MC3T3-E1 cells by regulating mitophagy through miR-29a-5p/PINK1 axis.

Bone regeneration is an important and sophisticated process involving the coordinated spatio-temporal regulation of a multitude of factors. In the past decade, the therapeutic potential of BMSC-Exos in bone regeneration continues to receive widespread attention [35,36], while as an essential exosomal cargo, the role of circRNAs in osteogenesis are often underserved and overlooked. In this research, we find that circHIPK3 is enriched in BMSC-Exos and promotes osteogenic differentiation of MC3T3-E1 cells by regulating mitophagy through miR-29a-5p/PINK1 axis (Figure 7). To the best of our knowledge, this is the first study demonstrating the pro-osteogenic function of exosomal circHIPK3, which provides a potential target for bone regeneration.

As a well-known cancer-associated circRNA, circHIPK3 is acknowledged to be relevant to many human diseases [37]. Recently, the role of circHIPK3 in osteogenesis has gradually garnered attention. Our results showed that BMSC-Exos treatment increased the expression of circHIPK3 (Figure 3C) and promoted the osteogenic differentiation of MC3T3-E1 cells by enhancing the mitophagy levels (Figure 5 and Figure 6). However, these results seem to be in contrast with a previous study reporting that knockdown of circHIPK3 promotes the osteogenic differentiation of human BMSCs through activating the autophagy flux [38]. We harbored the idea that this phenomenon might be attributed to several reasons. First, circHIPK3 may exert different functions in different cell types. Take the effect of circHIPK3 on cancer cells proliferation as an example. It is reported that circHIPK3 is upregulated in both colon carcinoma cells and gallbladder cancer cells. However, circHIPK3 presents inhibitory effect on the proliferation of colon carcinoma cells, and the promotional effect on gallbladder cancer cells [39,40]. Similarly, there is a great possibility that circHIPK3 plays an opposite role in BMSCs and MC3T3-E1 cells. Second, both studies found that circHIPK3 was upregulated during the osteogenic differentiation of hBMSCs, which confirmed the role of circHIPK3 in osteogenesis. Additionally, we took a step further to focus on the pro-osteogenic function of exosomal circHIPK3 on MC3T3-E1 cells, but not on its parent BMSCs. Therefore, the focuses of our research and the published work [38] are different, and the results are not contradictory.

Here, we addressed the ceRNA mechanism of circHIPK3 in regulating osteogenesis. Actually, circRNAs can exert their effects through multiple other mechanisms, such as competitively inhibiting its linear form transcription, coding protein or functioning as protein scaffolds [41]. In the current study, we detected the expression level of linear HIPK3 mRNA in MC3T3-E1 cells treated with BMSC-Exos and found no significant differences (Figure S3), suggesting circHIPK3 did not affect the expression of its linear form. We predicted the open reading forms (ORFs) of circHIPK3 by ORFfinder (https://www.ncbi.nlm.nih.gov/orffinder/ (accessed on 3 February 2014)) and found circHIPK3 contains at least 9 ORFs, suggesting the translation potential of circHIPK3. Additionally, four proteins (EIF4A3, IGF2BP1, IGF2BP2 and IGF2BP3) are predicted to bind with circHIPK3 by the CircInteractome database (https://circinteractome.nia.nih.gov/ (accessed on 1 February 2013)). Some scattered studies have touched upon the regulatory potential of these proteins during bone regeneration [42,43]. These predicted results suggest that circHIPK3 may regulate osteogenesis in complicated ways, which is worth exploring in the near future.

Mitophagy is a selective form of autophagy to remove the damaged mitochondria for maintaining mitochondrial quality [20]. Our previous work revealed that mitophagy promoted osteogenesis by transporting mineralization precursors from mitochondria to extracellular matrix [21]. Subsequently, Fei et al. found that PINK1/Parkin-dependent mitophagy was mediated by exosomes to regulate osteogenic differentiation of periodontal ligament stem cells [44]. However, little is known about the role of exosomal circRNAs in regulating mitophagy. Here, we highlighted the important role of mitophagy in osteogenesis in the current research. Moreover, we uncovered a possible molecular mechanism, the mitophagy level is regulated by circHIPK3/miR-29a-5p/PINK1 axis. Our study enriched the role of circHIPK3 and provided novel insights to understand the function of mitophagy during osteogenesis.

Admittedly, this study had some limitations. First, as mentioned before, circRNA exerts a variety of modes of action, such as inhibiting its linear form transcription, coding protein or functioning as protein scaffolds. In the present study, we focused on the ceRNA mechanism did not explore other possibilities. Second, it is possible that other BMSC-Exos cargos, in addition to circHIPK3, might be involved in the pro-osteogenic function, which is evidenced by the increased ALP activity and Alizarin Red S intensity of MC3T3-E1 cells treated by si-circHIPK3-BMSC-Exos (Figure 3E,F). However, we did not explore more detailed information. Therefore, in the next stage, we plan to investigate other mechanisms and the effect of other BMSC-Exos cargos in osteogenesis. Additionally, we attempt to produce engineering exosomes containing more bioactive circHIPK3 by gene-editing technology. With the help of biomaterials, we will develop a novel therapeutic strategy based on exosomal circHIPK3 for clinical bone regeneration.

## 4. Materials and Methods

### 4.1. Cell Culture

Primary mouse BMSCs cultures were prepared as described [45]. After aseptically removing femurs and tibiae, bone marrow was flushed from long bones of 8-week-old C57BL/6 mice with phosphate-buffered saline (PBS) using a sterile syringe. Bone marrow samples were centrifuged for 5 min at 1000× *g,* then placed in minimum essential α Modified Eagle Medium (α-MEM; Thermo Fisher Scientific Inc., Waltham, MA, USA) and replenished with fetal bovine serum (FBS, Gibco, Invitrogen, Waltham, MA, USA) and 1% penicillin and streptomycin (Solarbio, Beijing, China), then incubated at 37 °C with 5% CO_2_ for 48 h. Subsequently, the nonadherent cells were discarded, and the adherent cells were allowed to grow to approximately 80% confluence, which were defined as passage one cells (P1). At 70% confluence of P3 BMSCs, we exchanged the growth medium with the exosome-free medium prepared as described [46]. FBS was depleted of exosomes by ultracentrifugation at 100,000× *g* at 4 °C for 16 h (Beckman Coulter Avanti J-30I, Brea, CA, USA), then the supernatant of FBS was collected and filtered with a 0.22 μm filter (Millipore, Middlesex County, MA, USA). P3 BMSCs were used for all experiments.

The mouse osteogenic cell line, MC3T3-E1, was purchased from the American Type Culture Collection (https://www.atcc.org/ (accessed on 2 January 2023)) and cultured in exosome-free α-MEM (Thermo Fisher Scientific Inc., Waltham, MA, USA) with 10% exosome-free FBS (Gibco) and 1% penicillin and streptomycin (Solarbio) at 37 °C under 5% CO_2_.

### 4.2. Flow Cytometry Analysis

BMSCs surface markers were analyzed by flow cytometry. The cells were stained with: CD31 (ab9498, Abcam, Boston, MA, USA), CD34 (ab81289, Abcam, Boston, MA, USA), CD45 (ab40763, Abcam, Boston, MA, USA), CD44 (ab243894, Abcam, Boston, MA, USA), CD73 (ab202122, Abcam, Boston, MA, USA), CD90 (ab225, Abcam, Boston, MA, USA) and CD105 (ab221675, Abcam, Boston, MA, USA). Data were collected on a FACSCalibur (BD Bioscience, Franklin Lakes, NJ, USA) and analyzed using FlowJo software (Tree Star Inc., Ashland, OR, USA).

### 4.3. Exosome Isolation

Exosome isolation was prepared as described [47]. Before exosome isolation, BMSCs were cultured in the exosome-free medium to avoid interference from bovine exosomes. After 72 h, cell culture medium was collected, and exosomes were isolated from the supernatant by serial centrifugation of 300× *g* for 10 min, 2000× *g* for 15 min and 12,000× *g* for 30 min to remove floating cells and cellular debris. The supernatant was then passed through a 0.22-μm filter (Millipore, USA). The percolate was collected and centrifuged at 150,000× *g* for 90 min at 4 °C (Optima™XPN, Beckman Coulter) to obtain the exosome precipitate. The pellet was resuspended in 1 mL of sterile PBS and filtered through a 0.22-μm syringe-driven filter unit (Millipore). Finally, exosomes were stored in aliquots of 200 μL at −80 °C until being used.

### 4.4. Exosome Characterization

BMSC-Exos were observed by TEM [48]. Approximately 10 μL of exosomes were fixed in 3% (*w*/*v*) glutaraldehyde and 2% paraformaldehyde in a cacodylate buffer (pH 7.3) and incubated for 10 min at room temperature. The fixed exosomes were then applied to a continuous carbon grid and negatively stained with 2% uranyl acetate for 10 min. The HT7700 TEM (HITACHI, Tokyo, Japan) was used to examine exosome samples and obtain images.

NTA was performed to measure the size and the concentration of the isolated exosomes using Zetasizer Nano ZS90 (Malvern, PA, USA), according to the operating instructions. The exosomes were diluted in 1 mL PBS to be within the recommended concentration range, then the diluted exosomes were injected into the Zetasizer Nano ZS90 instrument (Malvern, PA, USA). Five 60 s videos were captured for each sample during flow mode (camera settings: slider shutter 890, slider gain 146). The videos were analyzed with the NTA 3.2 software (Malvern). All measurements were performed at room temperature.

Western blot was performed to detect the specific markers of exosomes (CD9, CD63, CD81 and TSG101) and endoplasmic reticulum (Calnexin) [49]. Briefly, proteins of exosomes and cells were measured by the Easy II Protein Quantitative Kit (BCA; TransGen Biotech Co., Beijing, China). Exosomes/cells were directly treated with protein loading buffer at 95 °C for 5 min, separated by SDS-PAGE, then transferred onto a polyvinylidene difluoride (PVDF; Millipore, Middlesex County, MA, USA) membrane. After blocking with 5% bovine serum albumin (BSA; Gibco) for 2 h, the membranes were incubated with primary antibodies at 4 °C overnight, then with horseradish peroxidase-conjugated goat-anti-rabbit secondary antibody at 37 °C for 1 h. Immunodetection was performed and quantified by chemiluminescence using an enhanced chemiluminescence assay kit (Amersham Pharmacia Biotech, Piscataway, NJ, USA) and Image Quant LAS 4000 (GE, Chicago, IL, USA). The CD9, CD63, CD81, TSG101 and Calnexin antibodies were purchased from Abcam.

### 4.5. Cellular Internalization of Exosomes

To observe the internalization of exosomes, BMSC-Exos and MC3T3-E1 cells were labeled by fluorescence dye CM-Dio (Thermo Fisher Scientific Inc., Waltham, MA, USA) and CM-Dil (Thermo Fisher Scientific Inc., Waltham, MA, USA), respectively, as previously described [50]. Briefly, 20 μg exosomes was diluted with 100 μL PBS and incubated with CM-Dio in the dark for 30 min, washed with PBS, ultracentrifuged at 120,000× *g* for 70 min to remove nonbinding dye, then resuspended in PBS. MC3T3-E1 cells were incubated with CM-Dil in the dark for 30 min and centrifuged at 300× *g* for 5 min to remove nonbinding dye. The CM-Dio labeled exosomes were incubated with CM-Dil-labeled MC3T3-E1 cells at 37 °C for 48 h. After the incubation, a fluorescence microscope (BX53, Olympus, Tokyo, Japan) was used to collect and analyze the fluorescence images.

### 4.6. Transwell Coculture

A Transwell (Corning, New York, NY, USA, 0.4 µm) coculture system was used in this study. The cells were categorized into four groups: MC3T3-E1 cells alone (the Control group), MC3T3-E1 cells cocultured with untreated BMSCs (the BMSC group), BMSC-Exos-treated MC3T3-E1 cells (the BMSC-Exos group) and MC3T3-E1 cells cocultured with GW4869 (Sigma-Aldrich, St. Louis, MO, USA, 10 μM) pretreated BMSCs (the BMSC + GW4869 group). Cells were cocultured for 1, 2, 3, 7 and 14 days and MC3T3-E1 cell proliferation and osteogenesis assays were subsequently performed. For the coculture experiment, exosomes (2 μg of exosomes per 100,000 recipient cells) were added to the culture medium.

### 4.7. Cell Proliferation

CCK-8 (Abcam) was used to determine the proliferation of MC3T3-E1 cells according to manufacturer’s instructions. Briefly, after coculturing in the Transwell for 1, 2, 3, 7 and 14 days, CCK-8 solution (100 μL) was added and incubated with MC3T3-E1 cells for 2 h at 37 °C. Subsequently, the absorbance of the formazan at 450 nm was measured using a microplate reader (Bio-Rad, Hercules, CA, USA) to evaluate MC3T3-E1 cell proliferation.

### 4.8. Quantitative Reverse Transcription-PCR (qRT-PCR)

Total RNA was extracted from BMSCs, BMSC-Exos and MC3T3-E1 cells using TRIzol reagent (Life Technologies, USA). Reverse transcription was performed using 1 μg of total RNA and the Fly First-Strand cDNA Synthesis SuperMix (TransGen Biotech Co.) according to the manufacturer’s protocol. An TransScript II Green One-Step qRT-PCR SuperMix (TransGen Biotech Co.) was used for qRT-PCR. The expression of GAPDH and U6 was used as controls to calibrate the original mRNA and miRNA concentrations in tissues and cells, respectively. Target gene expression was calculated using the 2^−ΔΔCT^ method. The primer sequences are detailed in Table S1.

### 4.9. Osteoblastic Differentiation Assays

The alkaline phosphatase (ALP) activity assay, Alizarin Red S (ARS) staining assays, qRT-PCR and Western blot were used to assess cell osteoblastic differentiation. MC3T3-E1 cells were cultured for up to 14 days in Transwell coculture system. 

As for the ALP assay, the cells were lysed with 1% Triton X-100 (Solarbio) at 37 °C for 2 h after culturing for 3, 7 and 14 days. ALP activity was evaluated via an ALP assay kit (Solarbio). In addition, a BCA protein assay kit (TransGen Biotech Co.) was used to obtain the ALP activity per unit protein by detecting the protein content. 

The ARS staining were conducted to assess calcium deposition. The cells were fixed with 4% formaldehyde for 20 min after culturing for 3, 7 and 14 days. Next, the cells were stained with 2% Alizarin Red (Solarbio, pH 4.2) for 10 min. Then, cetylpyridinium chloride (Solarbio) in 10% w/v 10 mM sodium phosphate (Solarbio, pH 7.0) was used for further quantitative analysis at 562 nm with a microplate reader. 

Regarding qRT-PCR, the cells were collected to extract the total RNA using TRIzol reagent (Life Technologies) after culturing for 14 days. Then, total RNA was converted into cDNA through the One-Step gDNA Removal and cDNA Synthesis SuperMix (TransGen Biotech Co.). Finally, TransScript II Green One-Step qRT-PCR SuperMix (TransGen Biotech Co.) was used to perform qRT-PCR. 

Western blot analysis was performed to detect osteogenic markers. In brief, whole cell were prepared using RIPA buffer (Solarbio). After electrophoresis, proteins were electroeluted onto a PVDF membrane (Millipore). Indicated primary antibodies were used. Protein bands were visualized by an enhanced chemiluminescence assay kit (Amersham Pharmacia Biotech). The following antibodies against RUNX2 (ab236639, Abcam), OCN (ab133612, Abcam) and GAPDH (ab8245, Abcam) were used. Each experiment was repeated at least three times with three replications per experiment.

### 4.10. PCR Amplification Using Divergent and Convergent Primers

To identify the loop structures of circRNA, PCR amplification was performed with the divergent and convergent primers (Table S1) by using cDNA and gDNA as templates. Divergent primers were used to flank the circRNA-specific junction (circular isoforms), while the convergent primers were used for the linear splice junction of downstream exons (linear isoforms). These primers were designed by an NCBI primer design tool and synthesized from Sangon Biotech (Shanghai, China) and 35 cycles of PCR were performed under standard conditions. The PCR products were then subjected to electrophoresis in 2% ethidium bromide-stained agarose gel. To confirm the PCR results, the PCR products were purified using an EasyPure Quick Gel Extraction Kit (TransGen Biotech Co.) and Sanger sequenced by Sangon Biotech (Shanghai, China).

### 4.11. RNase R Treatment

RNase R was used to detect the stability of RNA. A total of 10 µg RNA sample isolated from BMSCs using TRIzol reagent (Life Technologies) was mixed with 40 U RNase R (Epicentre Technologies Pvt. Ltd., Madison, WI, USA) for 15 min at 37 °C to remove the linear RNA and the levels of circHIPK3 and linear HIPK3 mRNA were examined by RT-qPCR.

### 4.12. Small Interference (si) RNA Transfection

Cells were transfected with siRNA reagents using X-tremeGENE siRNA Transfection Reagent (Roche, Switzerland) according to the manufacturer’s protocol. siRNA targeting circHIPK3 (si-circHIPK3-1 and si-circHIPK3-2) and matched negative control (si-NC) were synthesized from GenePharma (Shanghai, China). After transfection for 72 h, the expression of circHIPK3 was assessed by qRT-PCR. The sequences of si-circHIPK3-1, si-circHIPK3-2 and si-NC are set out in Table S2.

### 4.13. Dual-Luciferase Reporter Assay

The circHIPK3-WT, circHIPK3-Mut, PINK1-3′-untranslated region (UTR)-WT, and PINK1-3′-UTR-Mut were obtained by PCR. Sanger sequencing was performed to confirm the correct sequences. NotI/XhoI-digested psi-Check2 plasmid vector fragments were ligated into a NotI/XhoI-digested plasmid. Luciferase assays were performed using HEK293T cells. After being seeded into 96-well plates and reaching 50–70% confluency, the cells were transfected with psi-Check2-circHIPK3-WT (or psi-Check2-circHIPK3-Mut) or psi-Check2-PINK1-WT (or psi-Check2-PINK1-Mut) and the miR-29a-5p mimic or mimic-NC with Lipofectamine 3000 (Invitrogen). The cell lysates luciferase activity was measured after cells were transfected with the Dual-Luciferase System (Luciferase Assay Reagent, Promega, Madison, WI, USA) for 48 h.

### 4.14. Ago2-RIP Assay

Ago2-RIP was performed to detect the interaction between circHIPK3 and miR-29a-5p [51]. Briefly, cells were lysed in NP40 lysis buffer containing a protease inhibitor tablet (Roche) and RNA inhibitors. The Ago2-specific antibodies or mouse IgG control antibodies was used to immunoprecipitated cell lysate. The immunoprecipitated complexes were collected with Sepharose beads, washed three times with lysis buffer, incubated with RNase-free DNase I buffer and subjected to protease K (Solarbio) digestion. Coimmunoprecipitated RNAs were isolated and subjected to qRT-PCR analysis to detect the expression of circHIPK3 and miR-29a-5p.

### 4.15. CircRNA Pull-Down

Biotin-labeled circHIPK3 probe and control probe (Sangon Biotech, Shanghai, China) were used for circRNA pull-down assay as mentioned previously [48]. In brief, BMSCs were cross-linked by 1% formaldehyde for 30 min, lysed in co-IP buffer, and centrifugated. The supernatant was incubated with circHIPK3-specific probes-streptavidin beads (Sangon Biotech, Shanghai, China) mixture overnight at 37 °C. Then, the samples were washed and incubated with lysis buffer and proteinase K on the next day. Finally, the mixture was added with TRIzol reagent for RNA extraction and followed by detection of circHIPK3, miR-29a-5p, and GAPDH.

### 4.16. LC3 Immunofluorescence Staining

LC3 immunofluorescence staining was performed as described previously [52]. Briefly, after being seeded in glass coverslips and grown to required confluence, the cells were fixed with 4% paraformaldehyde (Solarbio) in PBS, permeabilized with 0.5% Triton X-100 (Solarbio) and blocked with 2% BSA (Gibco) in PBS. Cells were incubated with anti-LC3 antibody (1:100 in PBS-2% BSA; Abcam) overnight at 4 °C, washed thrice with PBS, incubated with secondary antibody (1:250) for 1 h and stained with Hoechst 33342 before mounting in glass slides and imaging in a confocal microscope (Olympus). ImageJ was used for quantification of LC3 puncta selecting the “Analyze Particles” function for ≥25 cells for each parameter.

### 4.17. Statistical Analysis

The SPSS 21.0 software was performed to the statistical analyses and the GraphPad Prism 6.0 (GraphPad Software, San Diego, CA, USA) was used to generate the graphs. Data are described as the mean ± SD. Student’s *t*-test was used to compare two groups and one-way analysis of variance (ANOVA) followed by Dunnett’s post hoc test, was used to compare more than two groups. Differences were considered statistically significant when *p* < 0.05.

## 5. Conclusions

The present study indicates that circHIPK3 is highly expressed in BMSC-Exos. Exosomal circHIPK3 promotes osteogenic differentiation of MC3T3-E1 cells through regulating PINK1 expression by sponging miR-29a-5p. The mitophagy level may be a downstream factor in mediating the impact of circHIPK3 on osteogenesis. Our study provides insights into the underlying mechanism of BMSC-Exos in the regulation of osteogenic differentiation.

## Data Availability

The datasets used or analyzed during the current study are available from the corresponding author upon reasonable request.

## Supplementary Materials

The following supporting information can be downloaded at: https://www.mdpi.com/article/10.3390/ijms24032785/s1.
