# Supplementary material for: BMSC-Derived Exosomal CircHIPK3 Promotes Osteogenic Differentiation of MC3T3-E1 Cells via Mitophagy
Source: Int J Mol Sci. 2023 Feb 1;24(3):2785. doi: 10.3390/ijms24032785 (PMC9917928; doi:10.3390/ijms24032785)
Supplement: Supplementary file 1 [file ijms-24-02785-s001.zip › Supplementary Figures S1-S3.pdf]

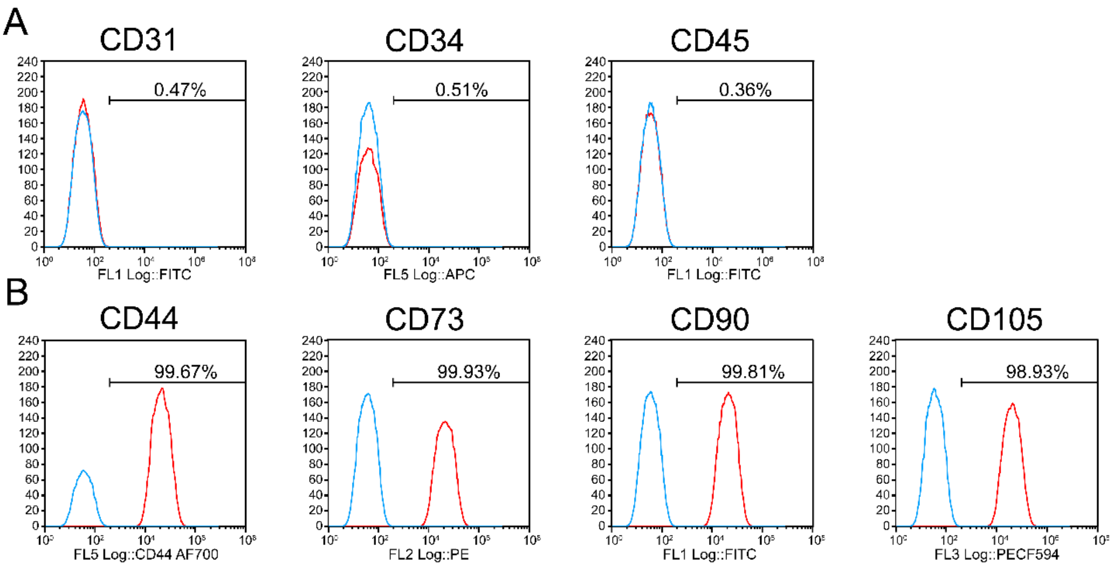

Figure S1. BMSC characterization using flow cytometry. The cells were positive for CD44, CD73, CD90, and CD105 (A) and negative for CD31, CD34 and CD45 (B). BMSC: Bone marrow mesenchymal stem cell.

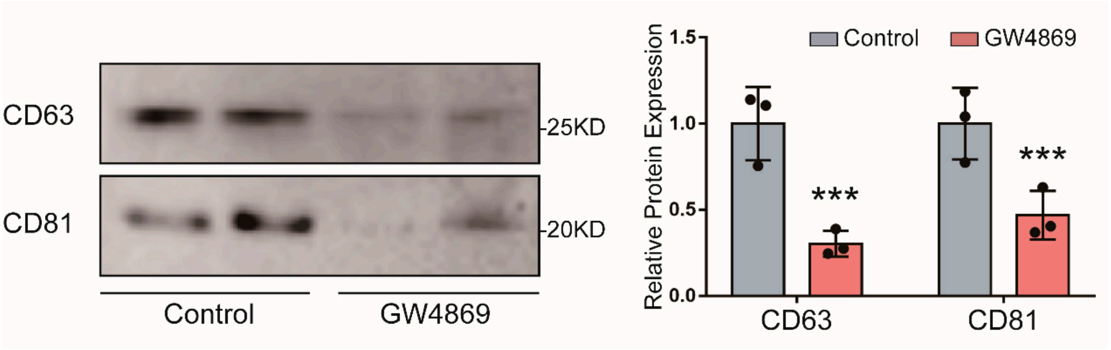

Figure S2. GW4869 administration inhibits exosome release. Seven days post GW4869 administration, exosomes were collected from supernatant of BMSCs. (A) and western blot (B) analyses. (n=3). *** p<0.001.

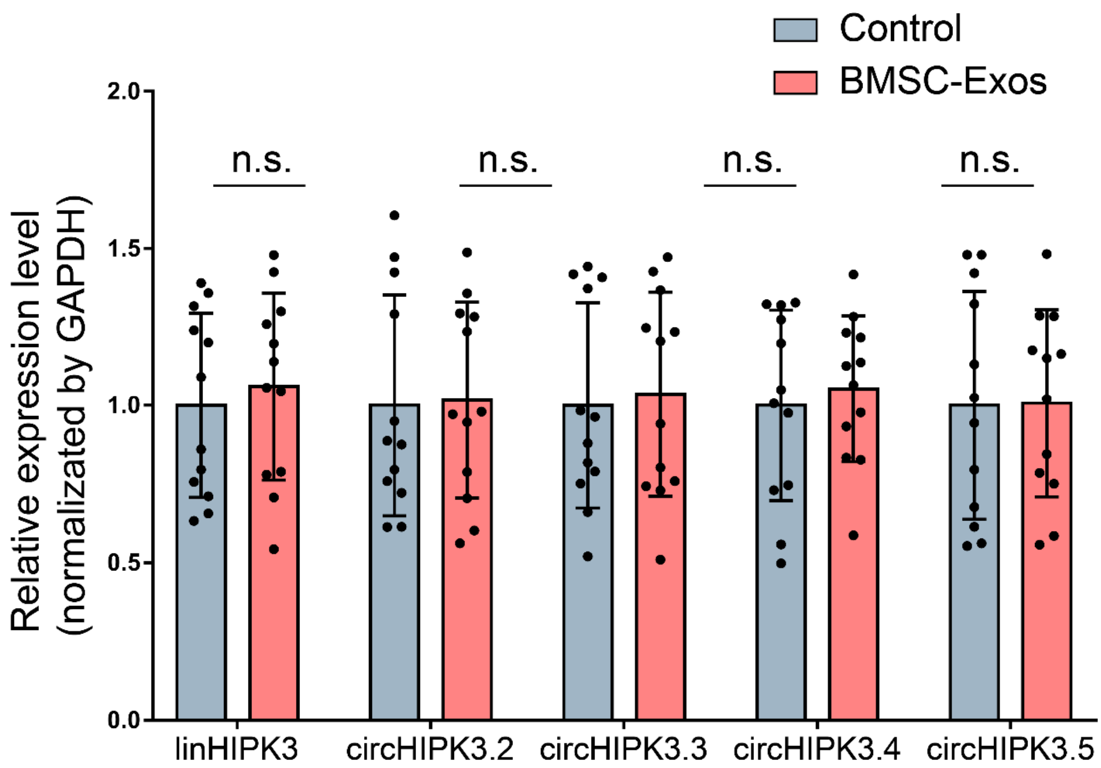

Figure S3. The relative expressions of linHIPK3 and the other homologous circRNAs of circHIPK3 in MC3T3-E1 cells treated with or without BMSC-Exos. *n*=12. *n.s.*, nonsignificant.
